# Are parenting behaviors associated with child sleep problems during treatment for acute lymphoblastic leukemia?

**DOI:** 10.1002/cam4.727

**Published:** 2016-04-25

**Authors:** Maria C. McCarthy, Jessica Bastiani, Lauren K. Williams

**Affiliations:** ^1^ Murdoch Children's Research Institute50 Flemington RoadParkvilleVictoria3052Australia; ^2^ Children's Cancer CentreRoyal Children's Hospital50 Flemington RoadParkvilleVictoria3052Australia; ^3^ Department of PsychologyUniversity of MelbourneParkvilleVictoria3010Australia

**Keywords:** Cancer, child sleep, leukemia, Parenting, pediatric oncology

## Abstract

Sleep disturbance is a recognized common side effect in children treated for acute lymphoblastic leukemia (ALL). Although associated with treatment factors such as hospitalization and corticosteroids, sleep problems may also be influenced by modifiable environmental factors such as parenting behaviors. The purpose of this study was to examine sleep problems in children undergoing treatment for ALL compared to healthy children and whether parenting practices are associated with sleep difficulties. Parents of 73 children aged 2–6 years who were (1) in the maintenance phase of ALL treatment (ALL group, *n* = 43) or (2) had no major medical illness (healthy control group, *n* = 30) participated in the study. Parents completed questionnaires measuring their child's sleep behavior and their own parenting practices. Parents of children undergoing ALL treatment reported significantly more child sleep problems; 48% of children with ALL compared to 23% of healthy children had clinical levels of sleep disturbance. Parents of the ALL group also reported significantly more lax parenting practices and strategies associated with their child's sleep including co‐sleeping, comforting activities, and offering food and drink in the bedroom. Results of multivariate regression analysis indicated that, after controlling for illness status, parent–child co‐sleeping was significantly associated with child sleep difficulties. Strategies employed by parents during ALL treatment may be a potential modifiable intervention target that could result in improved child sleep behaviors. Future research aimed at developing and testing parenting interventions aimed to improve child sleep in the context of oncology treatment is warranted.

## Introduction

Preschool children with acute lymphoblastic leukemia (ALL) have been shown to be at an increased risk of sleep problems 1, 2, 3, 4, with sleeping difficulties ranked by parents as the fourth highest somatic complaint in this population 5. Such difficulties may arise from the significant disruption to home and social environments associated with childhood cancer treatments 6. Additionally, side effects of medications such as corticosteroids that are administered for treatment of ALL, include sleep disturbance as well as alterations to emotional regulation and behavior 5, 7.

Sleep problems associated with children undergoing active treatment for ALL include increased sleep duration but also concerns around a lack of sleep in some children, higher sleep anxiety, parasomnias, greater daytime napping, disturbed sleep patterns, restlessness, pain, fatigue, and more night‐wakings, leading to increased fragmentation of sleep 3, 4, 7, 8, 9. Sleep difficulties may also occur after remission, with disturbances in night‐waking, temperature disturbance, nightmares, pain during sleeping, and other sleep difficulties, present in almost 50% of adult survivors of ALL 10.

Sleep disruption in healthy children is associated with increased impulsivity and hyperactivity, aggression, lower health‐related quality of life, behavioral concerns, as well as increased familial stress and poorer parent mental health 11, 12, 13. Although sleep is vital for healthy development in all children, it is even more important in children with serious and chronic illnesses, to help in growth and development, manage pain, and restore healthy body function 14. In preschool children being treated for ALL, sleep disruption has been associated with higher levels of anxiety and stress, and a lower perceived quality of life 1. The causes of sleep disturbance in young children treated for ALL is likely multifactorial and may be caused by side effects of corticosteroids and other drugs 7, 15, 16, and administration of medications throughout the night, thereby disrupting the child's sleep routine 16. Disruptions associated with hospitalizations including the interruption of everyday routines, family separations, and frequent night‐wakings for routine medical checks are also associated with child sleep disturbances 17, 18. Childhood sleep disturbance is also related to family environmental factors with sleep habits shaped by the parenting behaviors employed during early childhood development 19. This latter point is particularly relevant as the onset of ALL most commonly occurs in preschool‐aged children, a time when sleep patterns are strongly shaped by parental influences.

Studies to date indicate that, compared to parents of healthy children, parents of children with cancer may be more overprotective 20, more inconsistent in terms discipline 21, and more likely to spoil their child 21, 22, 23. While changes in parenting are likely associated with the family level challenges associated with childhood cancer and treatment 22, these parenting practices have been found to contribute to child behavioral problems in the general population 24. Few studies, however, have explored the role of parenting behaviors in predicting child sleep problems within the pediatric oncology context 5, 25.

In the general population, associations have been found between parental overprotection and child sleep problems 26, as well as between parental laxness in discipline and child sleep disturbance 24. Additionally, parental strategies for early sleep problems, including co‐sleeping and food and drink in the bedroom, may contribute to future sleep problems in healthy populations 27. However, a discernable gap in the literature exists concerning how parental overprotection, discipline, and behavioral strategies may impact on child sleep in pediatric oncology populations. Identifying whether parenting strategies influence children's sleep behaviors during cancer treatment is important as it offers a potentially modifiable target to improve child sleep and quality of life.

This exploratory study examined differences in child sleep problems and parenting strategies in families with a child with ALL compared to families of healthy children. A further aim was to explore whether parenting strategies were associated with child sleep problems, independent of medical factors. Study hypotheses were: (1) children with ALL will have more parent‐reported sleep problems than healthy children, (2) parents of children with ALL will report higher use of parenting behaviors associated with sleep problems, and (3) these parenting strategies will be associated with more child sleep problems independent of whether or not the child was receiving treatment for cancer.

## Method

### Participants

Participants were 73 parents of children aged 2–6 years who were either (1) in the maintenance phase of treatment for ALL (the “ALL” group) (*N* = 43) or (2) had no major medical history including developmental disorder, medical condition, or chronic illness (the “Healthy Control” group) (*N* = 30). Parents were not eligible for the study if they had insufficient English to complete questionnaires, if their child had relapsed or their cancer treatment was not considered curative or if their child had a major developmental disorder. The age range of 2–6 years was selected to reflect the most common period for presentation of childhood ALL and to ensure relative homogeneity of the group. Additionally, 2–6 years is a developmental period in which sleep behaviors and routines are commonly established and parenting strategies are highly influential.

### Procedure

This study was conducted in the Children's Cancer Centre (CCC) at The Royal Children's Hospital (RCH) Melbourne Australia, and received approval from The RCH Human Ethics Research Committee. Sixty‐nine families with children undergoing maintenance ALL therapy were identified during the recruitment period of which nine met exclusion criteria (insufficient English *N* = 4, relapsed *N* = 1, non curative *N* = 1, and developmental disorder *N* = 3). A total of 60 parents were approached for participation, 46 were approached in the clinic and 14 families who were not able to be seen in the clinic were contacted via mail. A total of six parents declined to participate and a further 11 consented but failed to return questionnaires, resulting in a final sample of *N* = 43 (response rate 71.7%). Reasons for non‐participation included lack of time, already participating in another research study, or due to reluctance to complete surveys generally. Patients with ALL were all treated on Children's Oncology Group protocols (ALL0331, ALL0232, ALL932, ALL0432).

Parents of children with ALL were also asked to provide contact details of other parents who had a healthy child of the same gender and age of their child. This recruitment approach was utilized in order to match the controls on key socio‐demographic characteristics. These identified parents were then contacted by telephone and asked to participate in the study. Nineteen parents provided details for 33 parent friends who had a healthy child aged 2–6 years (buddy controls). A total of 30 parents returned their surveys (90.9% response rate). Of note, over half of the ALL sample reported they could not provide contact details of potential healthy control participants, due to lack of contact with other parents with same‐aged children thus resulting in a smaller sample for this group 28.

### Materials

#### Tayside children's sleep questionnaire (TCSQ)

The TCSQ was the primary outcome measure for this study 29. The TCSQ is 10‐item scale in which parents rate the frequency with which their child exhibits various sleep behaviors using a 5‐point scale. An example item is “*The child has difficulty going to sleep at night (and may require a parent to be present)*”. The score range is 0–36 with higher scores indicating greater sleep disturbance and the diagnostic cutoff score is 8. The TCSQ is well validated and has good internal consistency (*a* = 0.85) 29; internal consistency in this study was adequate (*a *= 0.78).

#### Parenting protection scale (PPS)

The PPS is a 25‐item measure in which parents rate the extent to which each statement describes their behavior toward their child 23. This measure has previously been utilized to examine parental overprotection associated with childhood cancer 20. Example items include: “*I feed my child even if he/she can do it alone*”, “*I comfort my child immediately when he/she cries*”. Scores range 0–75 with higher scores reflecting a higher level of protective parenting behaviors. Criterion validity and internal consistency for this scale is good *α* = 0.73 23; for this study *α* = 0.70.

#### Arnold parenting scale (APS)

The APS is a self‐report measure of parental discipline 30. The APS has previously been utilized and validated in a large, Australian preschool sample 31. The 11‐item Laxness subscale of the APS short form 32 was used to assess parental laxness. Items are scored on a 7‐point Likert scale, and then averaged, with higher scores reflecting greater use of lax parenting (Range = 1–7). An example item is: “*I am the kind of parent that* (1) *sets limits on what my child is allowed to do* (2) *lets my child do whatever he/she wants*. Internal consistency for this subscale is good, *α* = 0.83 30; for this study *α* = 0.78.

#### Parental sleep strategies

Parent sleep strategies were assessed using items that were developed specifically for this study. Items were based on existing literature 9, consultation with a sleep expert, and clinical expertise of the research team. Parents are asked to rate the frequency upon which they used seven sleep strategies over the past 3 months to (1) get their child to sleep and (2) resettle their child midsleep. Items were rated on a 5‐point scale (*never, rarely, sometimes, often, always)*. The seven strategies were: *“Co‐sleeping (i.e., sleeping with the child for part/all of the night)”*, “*Food and drink in the bed or bedroom*”, *“Provision of bedtime routines”, “Use of controlled crying”*, “*Use of medication”, “Television in the bed or bedroom”,* and “*Comforting activities (e.g., rocking/holding the child, lying with your child to resettle them)”*.

#### Demographic characteristics

Demographic information including child age and gender, and parent education level was collected using a purposely designed questionnaire. Parents also reported date of their child's diagnosis, and treatment status; this data was verified from patient medical records.

### Statistical analyses

Statistical analyses were conducted using the statistical software packages SPSS version 21.0 (SPSS Inc., Chicago, IL). Descriptive statistics were calculated for all study measures. Two sample t‐tests and Pearson's Chi‐square tests were utilized to examine group differences on demographic, sleep behavior, and parenting strategies (Hypotheses 1 and 2). Univariate linear regressions were conducted to assess associations between demographic and parenting strategies and child sleep behavior which was measured as the total score on the TCSQ (Hypothesis 3). Variables significantly associated with the sleep outcome variable (*P* < 0.05) were included in a final, multivariate model. A hierarchical linear regression model was conducted with the group variable entered in Step 1 to account for whether or not the child was receiving cancer treatment and parenting variables entered in Step 2. This approach allowed for adjustment of the role of cancer‐related medical factors (i.e., via inclusion of the group variable) on child sleep behavior, and also for assessment of both the combined and unique contribution of each of the parenting variables.

## Results

Analysis of demographic characteristics of the two groups revealed there were significantly more males in the ALL group compared to the healthy control group (69.8% vs. 36.7%, *P *< 0.005). No other demographic differences were identified between the two groups. A summary of the demographic characteristics for the sample is provided in Table 1. With regard to child sleep behaviors, parents of the ALL group reported significantly more sleep disturbance for their children than the control group (ALL group mean** = **9.83, control group mean** = **5.73, *t *= 2.58, *P *= 0.012, 95% CIs = 0.93, 7.27). There was also a significant difference in the proportion of children scoring above the clinical cutoff for diagnostic sleep disturbance in the ALL group (47.6%) compared to the control group (23.3%) (*P *= 0.036).

**Table 1:** Overall sample characteristics and group differences in socio‐demographic characteristics, child sleep, and parenting strategies

	Total sample(*N *= 73)Mean (SD) or *N*(%)	CCC Group(*N *= 43)Mean (SD) or *N*(%)	Healthy control group(*N *= 30)Mean (SD) or *N*(%)	*P*‐value^a^
Socio‐demographic variables				
Child age (years)	4.73 (1.23)	4.60 (1.18)	4.92 (1.31)	0.284
Child gender				
Male	41 (56.16%)	30 (69.77%)	11 (36.67%)	0.005
Child months postdiagnosis	N/A	19.89 (6.76)^a^	N/A	N/A
Parent age (years)	36.23 (4.82)	36.03 (5.03)	36.51 (4.58)	0.680
Parent education				
High	37 (50.68%)	23 (53.49%)	14 (46.67%)	0.566
Number of children living at home	2.21 (0.84)	2.21 (0.89)	2.21 (0.79)	0.981
Marital status				
Married/De facto	67 (94.37%)	38 (90.48%)	29 (100.00%)	0.087
Child sleep problems				
Total score	8.13 (6.9)	9.83 (7.55)	5.73 (5.09)	0.012
At/above clinical cutoff b	27 (37.5)	20 (47.6%)	7 (23.3%)	0.036
Parenting strategies				
Parental overprotection	31.60 (6.37)	32.05 (6.92)	30.97 (5.57)	0.482
Parental laxness	2.69 (0.75)	2.93 (0.76)	2.35 (0.61)	0.001
Parent–child co‐sleeping^c^				
Sometimes or more	32 (43.83%)	23 (53.49%)	9 (30.00%)	0.039
Provision of food/drink in bedroom^c^				
Sometimes or more	19 (26.02%)	16 (37.20%)	3 (10.00%)	0.008
Comforting activities				
Sometimes or more^c^	31 (42.47%)	21 (58.1%)	10 (33.3%)	0.032

Differences in mean by group tested using two‐sample t‐tests. Pearson's Chi‐square tests used to examine associations between categorical variables.

a *N* = 4 (9.3%) 6–12mths postdiagnosis, *N* = 14 (32.6%) 12.01–18mths postdiagnosis, *N* = 16 (37.2%) 18.01–24mths postdiagnosis, *N* = 4 (9.3%) 24.01–30mths postdiagnosis, *N* = 5 (11.6%) 30.01–36mths postdiagnosis.

b Based on total difficulties cutoff score of disorders of initiating and maintaining sleep (≥8).

c Reference Never or Rarely.

Examination of parenting strategies revealed that parents of children with ALL reported significantly more lax parenting (ALL group mean** = **2.93, control group mean** = **2.35, *t* **= **3.426, *P* **= **0.001, 95% CIs** = **0.24, 0.91) (see Table 2) and more strategies for managing their child's sleep including, co‐sleeping (*P* **= **0.039), providing food and drink in the bedroom (*P* **= **0.008), and comforting activities (*P* **= **0.032). There was no difference detected between the groups on parental overprotection.

**Table 2:** Linear regression results of univariate associations between socio‐demographic and parenting variables with child sleep problems

Covariate	Total sleep difficulties score Coefficient (95% CI*)	*P*‐value	R^2^
Gender (reference category = male)	−2.587 (−5.820, 0.645)	0.115	0.035
Child age (years)	−0.791 (−2.112, 0.531)	0.237	0.020
Child months postdiagnosis(CCC group only)	−0.192 (−0.540, 0.155)	0.269	0.030
Parent age (years)	−0.197 (−0.539, 0.144)	0.254	0.019
Parent education (reference category=low)	−2.319 (−5.369, 1.092)	0.191	0.024
Number of children in household	0.736 (−1.266, 2.738)	0.466	0.008
Marital status(reference category = married/de facto)	5.076 (−2.015, 12.167)	0.158	0.029
Parental overprotection	0.217 (−0.042, 0.477)	0.100	0.039
Parental laxness	3.601 (1.54, 5.66)	0.001	0.152
Parent–child co‐sleeping	7.48 (4.72, 10.25)	<0.0001	0.294
Provision of food/drink in bedroom	3.34 (−0.29, 6.96)	0.071	0.046
Comforting activities	5.876 (2.916, 8.830)	<0.0001	0.183

CCC, Children's Cancer Centre.

* CI = Confidence Intervals

Univariate analyses revealed no significant association between demographic variables and parent‐reported child sleep behaviors (both ALL group and control group combined) (Table 2). Increased parental laxness (*β* = 3.601, *P *= 0.001), parent co‐sleeping (*β* = 7.48, *P *< 0.001), and parent comforting activities (*β* = 5.876, *P* < 0.001) were all positively associated with parent‐reported child sleep problems. Parental overprotection and provision of food and drink in the bedroom were not associated with child sleep problems.

A stepwise hierarchical regression was conducted with the group variable (ALL vs. healthy control group) entered first to account for any contribution of medical/illness factors. Illness status was significantly associated with child sleep problems, contributing to 7.8% of the variance (Adjusted *R* ^2^ = 0.078, *P* = 0.011). After controlling for illness status, the only parenting variable significantly related to child sleep problems was parent–child co‐sleeping (*R* ^2^ change = 0.299, *P *<* *0.001). Parent laxness and comforting activities did not significantly contribute to the final model (Table 3). Overall, the full model accounted for 35.3% (Adjusted *R* ^2^ = 0.353, *P*< 0.001) of the variance and significantly predicted child sleep problems. Parent–child co‐sleeping contributed 9.9% unique variance to the final model.

**Table 3:** Hierarchical linear regression examining associations between parenting strategies and sleep problems adjusting for illness status (*N *= 70)

Predictor variables	Adjusted R^2^	R^2^ change	t	*B*	*β*	*P*‐value
Step 1	0.078		4.47	0.302	4.27	0.011
Child illness status (reference category = healthy)						
Step 2	0.353	0.299				<0.0001
Child illness status (reference category = healthy)			1.01	0.108	1.52	0.316
Parental laxness			1.46	0.162	1.49	0.149
Parent–child co‐sleeping			3.26	0.374	5.24	0.002
Comforting activities			1.84	0.203	2.82	0.070

## Discussion

The purpose of this study was to investigate sleep problems in children aged 2–6 years undergoing maintenance phase treatment for ALL and, consistent with research to date, results indicated that parents of children in the ALL group reported significantly higher rates of child sleep problems than their healthy counterparts. Furthermore, the proportion of children in the ALL group scoring above the clinical cutoff indicating disorder of initiating or maintaining sleep (47.6%) is notably higher than the 35% prevalence found in the community‐based sample of more than 1000 children that was utilized to validate the TCSQ 29. The finding that almost half of parents of children on ALL maintenance therapy report clinically significant levels of child sleep problems, indicates that this is an important issue to target for intervention. In addition, parents of the children with ALL reported significantly more use of strategies to assist their child's sleeping, including co‐sleeping, use of comforting activities, and providing food and drinks in the bedroom. Given the well‐recognized family burden associated with childhood cancer diagnosis 6, including robust evidence for increased parental psychological distress 33, the additional efforts required to assist their child's sleeping may be particularly challenging for parents in this context 4.

Of the parenting strategies examined in this study, parent co‐sleeping emerged as the strongest factor associated with child sleep problems. This is consistent with the research literature examining sleep in healthy children that indicates co‐sleeping is often associated with poorer child sleep outcomes 27, 34. Given the cross‐sectional methodology of this study, it is not possible to attribute co‐sleeping as a casual factor in child sleep problems in this sample. For instance, co‐sleeping in the ALL group may result from parents attempting to ameliorate sleep disturbances associated with their child's illness. This hypothesis is supported by a qualitative component of this study that is reported elsewhere 25 and other studies in which parents reported either commencement or increase in co‐sleeping associated with hospitalizations, adapting to home post hospitalization and to comfort their child 4. Parents indicated that while co‐sleeping was intended as a short‐term measure, their children had become reliant upon it and, in some instances, it was associated with increased night‐waking 25. In another study conducted with children on maintenance phase treatment for ALL, 67% of parents reported their child's sleeping was altered postdiagnosis and this included more co‐sleeping and moving sleep locations during the night 9.

While not significant in the multivariate model, parent laxness was significantly associated with poor child sleep in the univariate analysis, a finding consistent with general child sleep literature 24. More lax parenting practices, generally characterized as less limit setting and discipline, may be an expected and even appropriate response to an unwell child, at least in the short term. However, the relationship between lax parenting and child psychosocial problems would suggest that this may be a modifiable target for healthcare providers to assist parents in improving child outcomes in the pediatric oncology context. An unexpected finding in the univariate analysis was that parents’ use of comforting behaviors (e.g., reading stories, rocking/holding) was associated with increased reported sleep problems. This is contrary to existing literature that suggests that these comforting measures are generally positively associated with child sleep patterns. It is possible that parents may be utilizing comforting activities as a response to their child's sleep disturbance or other behavioral disturbances such as separation anxiety. Alternatively, it is possible that within the ALL group, use of comforting activities is in the context of more lax parenting practices and increased co‐sleeping may contribute to an overall environment where the child is more reliant on their parents to actively assist them in either initiating or maintaining sleep. Given this is one of the first studies to examine parenting practices and child sleep problems in childhood cancer, further research is warranted to characterize these patterns.

The substantial level of reported sleep disturbance by parents of children with ALL and the evidence for parenting strategies to be associated with these difficulties support the notion that family‐based behavioral interventions may usefully be directed toward assisting families of young children with ALL. Within the general population, there is evidence that short‐term, targeted interventions for child sleep problems are beneficial 35. To date, there are no comparable interventions for families of children undergoing cancer treatment. Further research is required to establish whether generic or modified behavioral sleep interventions, may be useful for families of children undergoing cancer treatment 3. Given that child sleep problems are known to be influenced by additional environmental factors such as maternal mental health and specific illness/treatment factors such as the administration of corticosteroids, flexible implementation of evidence‐based strategies may be appropriate in the context of broader psychosocial supports for parents, patients, and families. In addition, the timing of psychosocial interventions aimed at improving child sleep need to be considered. Although the direction of the relationship between parenting practices and child sleep cannot be determined in the current cross‐sectional study, qualitative data suggests that strategies initially implemented by parents as short‐term measures to assist their child may become entrenched over time. It is possible that support provided to parents earlier in their child's treatment trajectory, including psychoeducation and direct consultations, may prevent longer term sleep problems in children undergoing cancer treatment.

The limitations of this study need to be considered. In particular, the small sample size necessitates that these findings be interpreted cautiously. Additionally, the study was conducted at one institution and thus the generalizability of the findings is limited. Employing buddy controls as a type of convenience sampling, also presents as a limitation, namely due to compromised external validity. This approach, however, enabled access to participants that were comparable in terms of sociodemographic characteristics, and also provided a fast, inexpensive, and readily accessible sample. The buddy control sample also maximized recruitment efforts, due to very high response rate (>90%) that would be much less likely in a general population. It is also notable that subgroup analyses revealed that parents who provided details of buddy control participants reported more child sleep problems than parents who did not provide contact details of buddy control participants. Although this finding should be viewed with caution given the small numbers, it is possible that those parents who reported child sleep difficulties were more motivated to participate in the study and therefore more willing to assist the research further by recommending others to the study.

The sample is also limited to young children, 2–6 years, treated for ALL. While this is also a strength of the study, providing a relating homogenous population at which to target interventions, the findings cannot necessarily be extrapolated to other cancer diagnoses or older children. The study also utilized a cross‐sectional methodology, as a result it is not possible to establish the directionality of the sleep associations. Future research utilizing longitudinal designs are needed to understand the direction of the relationship between parenting strategies and sleep disturbances. Further, the sleep problems identified in the study were determined by parent report and therefore susceptible to subjective factors including the potential to be influenced by parental stress associated with having a seriously ill child. In addition, there are no currently validated instruments designed to measure the specific sleep disruptions associated with childhood cancer treatments; development of such measures would be particularly useful for future research. Finally, more objective measures of child sleep including observational studies or use of technology devices such as actigraphy to measure sleep and sleep changes associated with particular oncology treatment cycles is warranted for future studies.

This study contributes to the small existing literature of increased sleep problems in young children undergoing treatment of leukemia and makes a significant novel contribution in identifying an association between parenting strategies and child sleep difficulties. For parents who are dealing with the challenges associated with childhood cancer treatment, child sleep difficulties may present a significant stressor in an already overburdened family system. The results of this study indicate that future research aimed at further delineating parenting practices and child sleep problems and the development of evidence‐based parenting interventions may be helpful in improving clinical and psychosocial outcomes for children undergoing cancer treatments and their families.

## Conflict of Interest

None declared.
